# Editorial to Special Issue “Cognitive Involvement in Multiple Sclerosis”

**DOI:** 10.3390/brainsci12050561

**Published:** 2022-04-27

**Authors:** Roberta Lanzillo

**Affiliations:** Department of Neurosciences, Reproductive and Odontostomatological Sciences, Federico II University of Naples, 80121 Naples, Italy; roberta.lanzillo@unina.it

Multiple sclerosis (MS) is a multifaceted and complex disorder that mainly affects young adults, impacting their work and social abilities. Both physical and cognitive disabilities are drivers for a low quality of life (QoL) in people with MS (pwMS). The prevalence of cognitive impairment (CI) in MS ranges from 34% to 65%, depending on both disease duration and age at disease onset. Moreover, this prevalence is influenced by how CI is defined, as this varies according to the number of neuropsychological batteries used and the definition of CI. Various aspects, such as disease phenotype (relapsing or progressive), fatigue, depression, extent of brain tissue damage, as well as motor-cognitive reserve, have been implicated to play a substantial role in the overall cognitive performances decline. It is noteworthy that the accumulating evidence reports that pwMS experience deficit in the less explored cognitive domains, such as theory of mind, pragmatics, meta-cognition and prospective memory etc, which might be affected in the absence of overall CI. Recently, diverse computerized neuropsychological testing tools have allowed for the application of more comprehensive assessment batteries. Yet, their application remains rather limited, due to the lack of reliability in studies with larger sample sizes.

To date, pathological brain changes associated with CI in MS are not fully understood. The application of novel advanced imaging techniques revealed the neural underpinnings of both the overall CI and the impairment in selected cognitive domains. Cortical and subcortical grey matter atrophy, as well as fiber tract interruption, functional changes and, hence, synaptic dysfunction, were found to have variable contribution to CI in both cross-sectional and longitudinal assessments. However, efficient approaches for treating CI are still lacking. Despite the efficacy of disease modifying treatment in preventing cognitive decline, clinical trials using effective molecules in other neurodegenerative disorders have been disappointing. Interestingly, cognitive neurorehabilitation in MS, through either conventional neuropsychological approaches or computerized neuropsychological training and serious video games, have provided promising effects.

This research topic was aimed at shedding further light on CI in MS and to unveil the pathological changes underpinning cognitive disability.

Authors have contributed with seven valuable works on different aspects of CI in MS, including two reviews on pediatric aspects and on neuroradiological correlates of CI in MS and five original papers.

These shed light on several aspects of CI in MS, ranging from etiopathogenesis, biomarkers and its assessment and prevention.

In particular, Virgilio and colleagues [1] investigated the role of vitamin D deficiency in CI in MS, especially regarding information processing speed (IPS). A high proportion of MS patients included in the study (87%) showed hypovitaminosis D and 23% had CI. The patients with CI showed severe hypovitaminosis D, while no patients with sufficient vitamin D levels had CI. Moreover, they found a positive correlation between the vitamin D levels at diagnosis and CI scores, which persisted after correction for sunlight exposure, MRI baseline characteristics and disability after a mean 2 year follow-up, suggesting that low vitamin D levels may affect both cognition and early disability in newly diagnosed MS patients.

Similarly, Reia and collegues [2] investigated the association between cardiovascular risk and neuropsychological performances in MS in a retrospective study, through the Framingham risk score, which provides the 10-year probability of developing macrovascular disease. They found that each point increase in the Framingham risk score corresponded to a lower score at the verbal learning test, particularly when examining the Framingham risk score components of sex and total cholesterol levels, with male gender and high lipid level related to lower cognitive scores. The authors, therefore, suggested that the effect of lifestyle and pharmacological interventions on cardiovascular risk factors should be considered in the management of MS, given the possible effects on cognitive function.

Switching to papers related to possible CI biomarkers in MS, in the interesting work by Paolicelli and colleagues [3], they performed a pilot study, applying magnetoencephalography (MEG) and high density (hd) electroencephalography (EEG) to evaluate the acoustic P300 features in a cohort of early MS patients. CI was assessed using Rao’s Brief Repeatable Battery (BRB). In pwMS, they observed a latency prolongation of a P300 peak compared to HCs, and an inverse correlation between the P300 amplitude and fatigue. The authors concluded that in pwMS, the phenomena of cortical adaptation to early dysfunction could preserve the cognitive performance of the P300 acoustic task, while the development of fatigue could prospectively lead to amplitude decline in P300, suggesting its possible role as a biomarker.

Govindarajan and colleagues [4] focused instead on advanced MRI techniques and their relation to the attention components, measured using the Attention Network Test for Interactions (ANT-I). They investigated gray matter through cortical thickness changes and deep gray matter through volumetric changes in young MS participants with pediatric or young-adult onset and mild disease severity. The thalamic volumes were significantly lower in MS participants. The slowed reaction times of the alerting component in MS correlated significantly with the reduced volume of the right pallidum in MS, while slowed reaction times of the executive control component correlated significantly with the reduced thickness in cortical areas and with reduced volume of the left putamen in MS (Figure 1).

These findings demonstrated an intriguing association between the gray matter changes and attentional performance early in the disease process and in patients without impaired attentional processes. Therefore, subcortical gray matter volume atrophy and cortical thickness changes could be considered as an early marker of MS pathophysiology, prior to cognitive deficit onset.

Eventually, Pitterri and colleagues [5] studied the extent to which the slowing in information processing speed (IPS), considered the key cognitive deficit in MS, can be affected by increased task demands. They developed three tasks, delivered with a tablet-based videogame. A significantly reduced performance of the pwMS, as compared with HC, was found on the videogame tasks, with the pwMS being on average slower and less accurate than HC. Furthermore, the pwMS showed a significantly more pronounced decrement in accuracy as a function of the visual attentional load, suggesting a higher susceptibility to increased task demands. The correlations provided evidence for the validity of the videogame as a valid tool to test IPS in pwMS, suggesting a high potential of computerized assessment tools in clinical practice.

The two interesting reviews are valuable tools to refresh our knowledge on two main aspects of MS.

In particular, Portaccio and collegues [6] clarified that pediatric patients are particularly susceptible to cognitive impairment, resulting from both disease-related damage and failure of age-expected brain growth. Cognitive impairment has, indeed, been consistently reported in approximately one-third of pediatric patients with MS, despite the lack of a uniform definition of cognitive impairment and the adoption of different tests. Moreover, research aimed at the identification of the risk factors (e.g., demographic, clinical, and radiological features) or protective factors (e.g., cognitive reserve and leisure activities) for cognitive decline is still scanty and mood disorders can further affect quality of life, as well as academic performances, in young pwMS. Portaccio et al., in their review, focused also on MRI features and attributed CI both to the damage of specific brain compartments and to abnormal brain network activation patterns. Importantly, longitudinal studies have recently demonstrated that the failure of age-expected brain growth and of white matter (WM) and gray matter (GM) maturation plays a relevant role in determining cognitive dysfunction, in addition to MS-related direct damage.

Lastly, Petracca and colleagues [7] described the MRI aspects of CI, offering an up-to-date overview of the latest findings on the structural, functional and metabolic correlates of CI in adults with MS, focusing on the mechanisms sustaining damage accrual and on the identification of useful imaging markers of cognitive decline.

In recent years, the development of new MRI sequences and modelling approaches has allowed the characterization of many structural and functional correlates of cognitive impairment in MS, providing new tools for disease monitoring and identifying new potential therapeutic targets. Although many questions remain unanswered, the value of advanced neuroimaging as an investigative tool of pathological changes in vivo remains undisputed, and future developments in this field will steadily add to our understanding of cognitive involvement in MS.

## Figures and Tables

**Figure 1:**
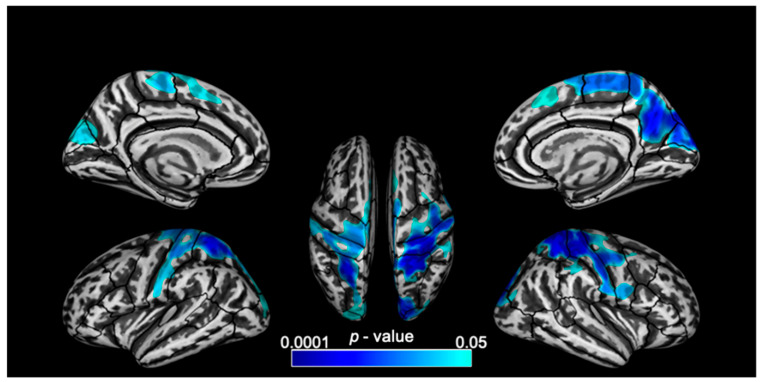
Lateral, medial and superior views of the brain with clusters of significant (*p* < 0.05, corrected for multiple parisons) negative correlation between cortical thickness and EXE scores (from the Attention Network Test for Interaction) comparisons) and negative correlation between cortical thickness and EXE scores (from the Attention Network Test for Interaction) overlaid on inflated surfaces.
